# Clinical Importance of Differentiating Epstein-Barr Virus (EBV)-Positive Plasmacytoma From Plasmablastic Lymphoma: Another Unique Case of EBV-Positive Plasmacytoma in an Immunocompetent Patient

**DOI:** 10.7759/cureus.40021

**Published:** 2023-06-06

**Authors:** Adrian M Alonso, Alyssa S Saxton, Rick Y Lin, Eric J Basile, Yu Yang

**Affiliations:** 1 Internal Medicine, University of Florida College of Medicine, Gainesville, USA; 2 Pathology, Immunology, and Laboratory Medicine, University of Florida College of Medicine, Gainesville, USA

**Keywords:** immuno-competent, hiv, plasmablastic large b-cell lymphoma, ebv associated lymphoma, solitary bone plasmacytoma

## Abstract

Epstein-Barr virus (EBV)-positive plasmacytoma is a rare and unique plasma cell neoplasm that could arise in immunocompetent individuals. Given the molecular and immunohistochemical similarity of EBV-positive plasmacytomas to their significantly more aggressive counterpart, plasmablastic lymphoma (PBL), providers must distinguish between the two neoplasms. This case elucidates a presentation of EBV-positive plasmacytomas in a healthy, immunocompetent individual originating in the C4/C5 cervical neck region. The patient's clinical presentation, in combination with the surgical pathology from the mass biopsy, pointed toward EBV-positive plasmacytoma. Factors such as cellular proliferation rate, cellular atypia, and immunohistochemical staining help differentiate the two diseases. This case will further help providers in the oncologic world to identify these masses.

## Introduction

Epstein-Barr virus (EBV) is a known oncogenic virus that has been the causative agent for various malignancies, including Burkitt's lymphoma, Hodgkin's lymphoma, and gastric carcinoma subtype [1]. Occasionally, EBV can contribute to the development of plasma cell-specific neoplasms, such as plasmablastic lymphoma (PBL), an aggressive lymphoma with poor outcomes, and plasmacytoma, a less invasive tumor with a better overall outlook. EBV positivity can occur in about 60-70% of patients with PBL, particularly those who are immunodeficient [2]. Plasmacytomas, however, rarely return positive for EBV, with only a few cases reported annually [3]. A positive EBV finding complicates the overall diagnosis as both diseases may have overlapping morphological and pathological similarities. Therefore, these two disease processes must be differentiated as the therapies used differ, as well as the prognosis [4]. Here, we present a patient with an EBV-positive plasmacytoma arising from the C4/C5 cervical neck region. 

## Case presentation

**Figure 1:**
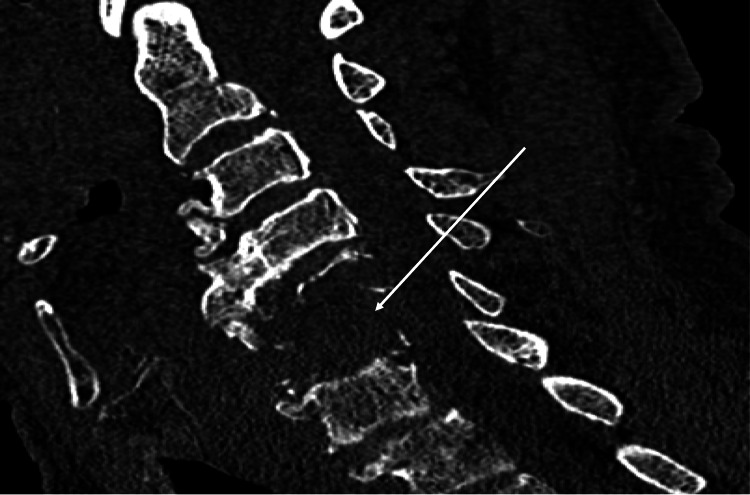
C5/C6 lesion (white arrow) CT scan depicting C5/C6 cervical bones with aggressive soft tissue mass causing complete destruction of the fifth and sixth cervical vertebra

**Figure 2:**
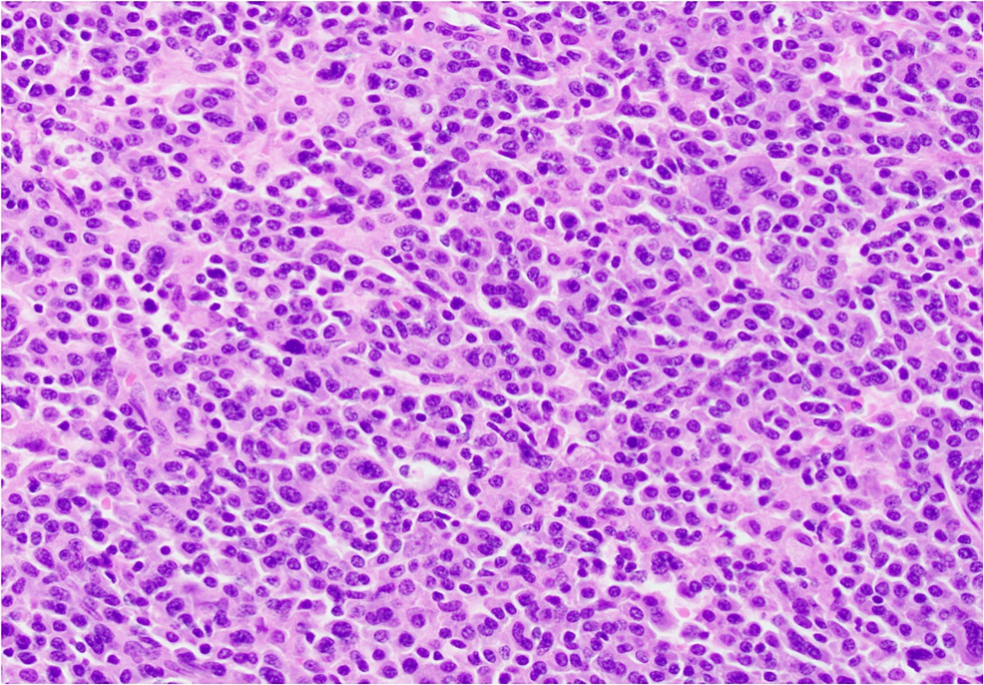
Hematoxylin and eosin-stained section Sheets of plasmacytoid cells without significant pleomorphism (200x)

**Figure 3:**
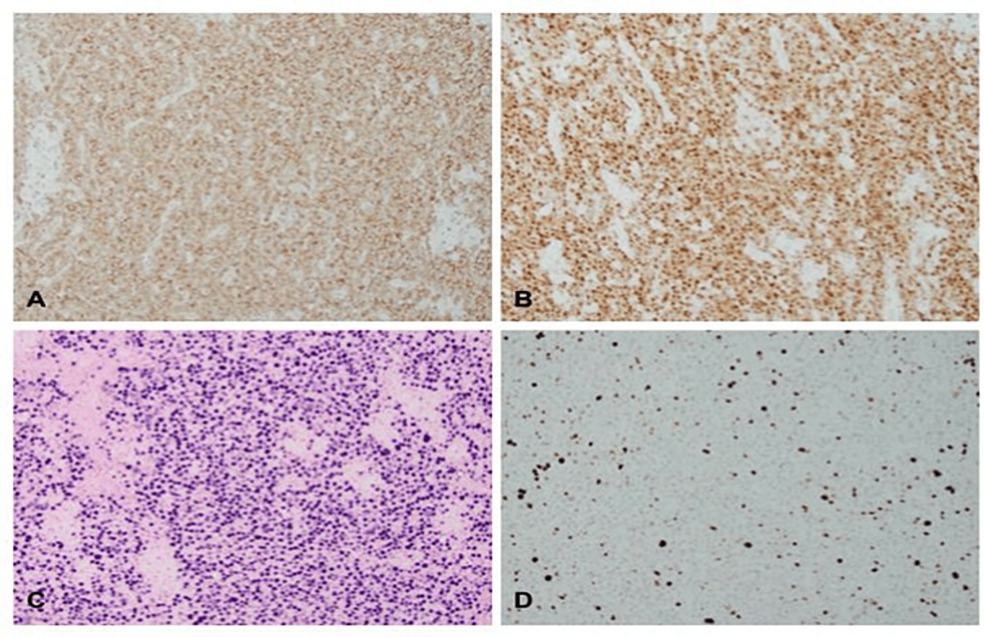
Immunohistochemical (IHC) studies Plasmacytoid cells are positive for CD138 (A) and MUM1 (B), and diffusely positive for EBER (C) by in-situ hybridization with relatively low ki-67 (D) proliferative index (<10%). All IHC stains: 200× EBER: Epstein-Barr encoding region; MUM1: multiple myeloma oncogene-1

A 54-year-old male with no significant past medical history besides tobacco use was admitted for ongoing cervical neck pain and myelopathy. Vitals on admission were within normal limits except for some tachycardia. CT cervical spine (C-spine) on admission depicted an aggressive-appearing soft tissue mass in the C5/C6 vertebra, causing complete destruction of the fifth and sixth cervical vertebra (Figure 1). He underwent an uncomplicated C5/C6 corpectomy with C4-C7 plating, and the mass was sent to pathology. The official pathology reading depicted EBV-positive plasma cell neoplasm with plasmacytoid cells (Figure 2). The further immunohistochemical analysis resulted in CD138 +, multiple myeloma oncogene-1 (MUM-1) +, and Epstein-Barr encoding region (EBER) positivity with a Ki-67 proliferation rate of less than 10% (Figure 3), with lambda restriction. There was an initial concern for multiple myeloma. However, this was later discarded as the patient did not have other laboratory signs of CRAB (calcium elevation, renal insufficiency, anemia, bone abnormalities) symptoms. The kappa/lambda ratio was within normal limits, and the CT multiple myeloma scan showed no further evidence of osteolytic lesions. The bone marrow biopsy was largely unremarkable and did not depict plasma cell neoplasms. Because of the lack of other systemic signs that would suggest multiple myeloma, the leading differentials were PBL or plasmacytoma. Because of the immunohistochemical findings (i.e., low Ki-67), his normal bone marrow and flow cytometry results, and his clinical history (negative HIV, no immunosuppression), plasmacytoma was higher on the differential. The patient was promptly set up with radiation oncology to undergo 10 sessions of radiation treatment. 

## Discussion

This case depicts another unique presentation of EBV-positive plasmacytoma in an immunocompetent individual. Plasmacytomas are rarely EBV-positive, especially in patients with no history of immune deficiency. To accentuate the rarity of this pathology, MD Anderson Cancer Center recently analyzed EBV-positive immunocompetent patients in their database and identified only four cases [2]. The patients were of various ages in all four cases, with asymptomatic presentations, and were immunocompetent. Similarly, the patient in this current report had clinical features similar to those mentioned above, and his presentation will add to the database of this unique disease. This report accentuates the clinical importance of correctly diagnosing PBL vs. plasmacytoma.

Plasmacytomas generally have morphological features which overlap with PBL, such as cellular atypia, with some cells appearing plasmablastic and, of course, EBV positivity. However, some pathological differences can help differentiate the two disease states. Plasmacytomas usually have a low Ki67 proliferation rate with less atypia, while PBLs have a high Ki67, with most cases being greater than 70%. To further appreciate this difference, the cases reported at MD Anderson had an average Ki67 proliferation rate of 25% for plasmacytomas [2]. MYC gene rearrangements, a known proto-oncogene, are also essential immunohistochemical signs for differentiation [5]. PBL overexpresses MYC aberrations with greater than 40% expressivity, especially in EBV-positive PBL, while plasmacytomas have low expression of MYC rearrangements. In addition, EBV-positive plasmacytoma is less commonly associated with the quintessential "starry sky" appearance and tends to have light chain restriction and no necrosis [4,6,7].

Our patient met many criteria suggesting EBV-positive plasmacytoma, including low Ki67, light chain restriction, no necrosis, and, as mentioned before, immunocompetent status. Plasmacytomas are relatively indolent; treatment usually comprises radiation therapy or minimal chemotherapy [4]. PBL, on the other hand, is a dismal disease with a poor prognosis and few evidence-based treatments. Treatments usually involve CHOP (cyclophosphamide, doxorubicin, vincristine, prednisone) or EPOCH (etoposide, vincristine, doxorubicin, cyclophosphamide, and prednisone) therapies [8]. In addition, it is essential to catch plasmacytomas early, as some cases have found that these masses can progress to PBLs [9]. 

## Conclusions

Given the rarity of EBV-positive plasmacytomas, this report adds another case to the body of growing medical research literature about this unique disease. This report aims to augment the reported cases of EBV-positive plasmacytomas and emphasize the importance of differentiating between PBL and EBV-positive plasmacytoma. More such cases need to be reported in order to increase provider suspicion of plasma cell neoplasm in the presence of EBV positivity. 

## References

[1] Bakkalci D, Jia Y, Winter JR, Lewis JE, Taylor GS, Stagg HR (2020). Risk factors for Epstein Barr virus-associated cancers: a systematic review, critical appraisal, and mapping of the epidemiological evidence. J Glob Health.

[2] Loghavi S, Khoury JD, Medeiros LJ (2015). Epstein-Barr virus-positive plasmacytoma in immunocompetent patients. Histopathology.

[3] He L, Li Z, Fan X, Shi Q, Wu H, Chen J (2020). Epstein-Barr virus-positive plasmacytoma in immunocompetent patients: a diagnostic dilemma. Int J Clin Exp Pathol.

[4] Zhou T, Cheng J, Karrs J (2022). Clinicopathologic and molecular characterization of Epstein-Barr virus-positive plasmacytoma. Am J Surg Pathol.

[5] Casey SC, Baylot V, Felsher DW (2018). The MYC oncogene is a global regulator of the immune response. Blood.

[6] Gibbs JD, Leon ME, Liu K, Nguyen J, Zhang L (2017). A rare case of Epstein-Barr virus‐related plasmacytoma involving maxillary sinus mucosa. Clin Case Rep.

[7] Valera A, Balagué O, Colomo L (2010). IG/MYC rearrangements are the main cytogenetic alteration in plasmablastic lymphomas. Am J Surg Pathol.

[8] Castillo JJ, Bibas M, Miranda RN (2015). The biology and treatment of plasmablastic lymphoma. Blood.

[9] Ambrosio MR, De Falco G, Gozzetti A (2014). Plasmablastic transformation of a pre-existing plasmacytoma: a possible role for reactivation of Epstein Barr virus infection. Haematologica.

